# Navigating the Metabolic-Genomic Paradigm: Mitochondrial Reprogramming as a Driver of Cancer Plasticity

**DOI:** 10.32604/or.2026.078924

**Published:** 2026-07-16

**Authors:** Yen-Dun Tony Tzeng, Chen-Yueh Wen, Su-Boon Yong, Zhi-Hong Wen, An-Jen Chiang, Chia-Jung Li

**Affiliations:** 1 Department of Surgery, Kaohsiung Veterans General Hospital, Kaohsiung, Taiwan; 2 Center of General Education, Shu-Zen Junior College of Medicine and Management, Kaohsiung, Taiwan; 3 Division of Urology, Show Chwan Memorial Hospital, Changhua, Taiwan; 4 Division of Urology, Chang Bing Show Chwan Memorial Hospital, Changhua, Taiwan; 5 Department of Allergy and Immunology, China Medical University Children’s Hospital, Taichung, Taiwan; 6 Research Center for Allergy, Immunology, and Microbiome (A.I.M.), China Medical University Hospital, Taichung, Taiwan; 7 Department of Marine Biotechnology and Resources, National Sun Yat-sen University, Kaohsiung, Taiwan; 8 National Museum of Marine Biology & Aquarium, Pingtung, Taiwan; 9 Department of Medical Education and Research, Kaohsiung Veterans General Hospital Kaohsiung, Kaohsiung, Taiwan; 10 Department of Obstetrics and Gynecology, Kaohsiung Veterans General Hospital, Kaohsiung, Taiwan; 11 Center of General Education, Cheng Shiu University, Kaohsiung, Taiwan; 12 Institute of BioPharmaceutical Sciences, National Sun Yat-sen University, Kaohsiung, Taiwan

**Keywords:** Breast cancer (BC), precision medicine, metabolic reprogramming, therapeutic resistance, artificial intelligence (AI)

## Abstract

Breast cancer (BC) management has transitioned from histological classification to molecular subtyping, yet therapeutic resistance and intratumor heterogeneity remain critical clinical challenges. This review examines the emerging paradigm shift toward integrating mitochondrial metabolism into the precision medicine framework. We detail the complex mitonuclear crosstalk where nuclear genetic alterations, such as Breast Cancer 1 (*BRCA1*) deficiency and *TP53* mutations, fundamentally reprogram mitochondrial bioenergetics. Specifically, the loss of BRCA1 function triggers a systemic NAD^+^ depletion trap through *PARP1* hyperactivation, while oncogenic drivers like *MYC* coordinate with *PGC1α* to enhance mitochondrial biogenesis for metastatic survival. We evaluate the diagnostic potential of mitochondrial DNA heteroplasmy and machine learning derived metabolic gene signatures as high performance biomarkers for patient stratification and the detection of minimal residual disease via liquid biopsy. Furthermore, we analyze current clinical efforts to target mitochondrial vulnerabilities, including respiratory chain inhibitors like metformin and BH3 mimetics, while highlighting the significant challenges posed by metabolic plasticity and nutrient competition in the tumor microenvironment. The analysis of clinical trial data, such as the MA.32 study, suggests that metabolic interventions require precise patient selection based on specific metabolic phenotypes rather than broad application. Looking forward, the integration of genome scale metabolic models and artificial intelligence (AI) offers a transformative pathway to simulate patient specific metabolic fluxes and identify novel synthetic lethal targets. By bridging the gap between nuclear genomic drivers and dynamic mitochondrial adaptations, this review aims to provide a preliminary framework for the exploration of metabolic-genomic precision oncology in BC.

## 1 Introduction

The clinical management of breast cancer (BC) has undergone a fundamental transformation over the past several decades, evolving from a reliance on anatomical parameters to a sophisticated framework of molecular precision [1]. Historically, the Tumor-Node-Metastasis (TNM) staging system served as the primary arbiter of treatment decisions. However, the advent of high-throughput technologies has enabled a transition toward intrinsic molecular subtyping—classifying tumors into Luminal A, Luminal B, HER2-enriched, and Triple-Negative Breast Cancer (TNBC) based on gene expression profiles and receptor status [2,3]. While these genomic classifications have significantly improved the personalization of adjuvant therapies, BC remains a leading cause of female mortality globally due to its profound intra-tumor heterogeneity and the adaptive capacity of cancer cells to evade standard-of-care treatments [4,5]. Current genomic assays, such as Oncotype DX and MammaPrint, offer predictive value for recurrence, yet they often fail to account for the dynamic metabolic adaptations that occur during therapy, highlighting the need for a more multidimensional approach to precision medicine [6]. 

This clinical gap has catalyzed a “metabolic paradigm shift,” where the focus has broadened from purely genetic aberrations to the complex rewiring of cellular bioenergetics [7]. Metabolic reprogramming is now firmly established as a hallmark of malignancy, no longer viewed as a secondary byproduct of oncogenic signaling but as a primary driver of tumor progression and therapeutic resistance [8,9,10,11]. Within this landscape, mitochondria have emerged as central integrative hubs. In contrast to the classical Warburg hypothesis suggesting that cancer cells rely almost exclusively on aerobic glycolysis due to defective mitochondria, recent evidence demonstrates that mitochondria in most BCs are highly functional and versatile [12]. These organelles facilitate metabolic plasticity, allowing cancer cells to switch between glycolysis and oxidative phosphorylation (OXPHOS) to survive in nutrient-deprived or hypoxic microenvironments [8,12]. Crucially, the “mitochondrial phenotype” of a tumor has been shown to hold significant prognostic value. Aggressive BC subtypes often exhibit an increase in mitochondrial biogenesis and mass, which supports the high energetic demands of rapid proliferation and metastatic colonization [13,14]. High expression of mitochondrial markers, such as the Translocase of Outer Mitochondrial Membrane 20 (*TOMM20*), has been validated as an independent predictor of poor overall survival and resistance to chemotherapy across multiple BC cohorts [15,16]. Furthermore, a mitochondrion-rich phenotype is frequently associated with advanced tumor stages, high Bloom-Richardson-Elston (BRE) grades, and a higher propensity for nodal metastasis [13]. These findings suggest that quantifying mitochondrial activity provides a critical dimension of tumor biology that current genomic subtyping may overlook [16].

**Figure 1:**
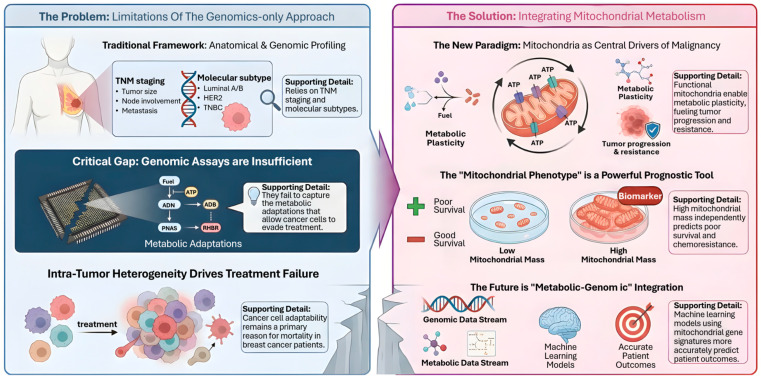
**The transition from genomics-only clinical frameworks to a metabolic-genomic integrated paradigm in breast cancer (BC).** Left Panel: The Problem—Limitations of the Genomics-only Approach. Traditional clinical frameworks primarily rely on TNM staging (Tumor size, Node involvement, and Metastasis) and Molecular Subtyping (e.g., Luminal A/B, HER2^+^, TNBC) for prognosis. However, these genomic assays are insufficient as they fail to capture dynamic Metabolic Adaptations and the complexity of Intra-tumor Heterogeneity. This diagnostic gap often leads to treatment failure because a subset of cancer cells can adapt their metabolic pathways to evade therapy. Abb: TNM, Tumor-Node-Metastasis; TNBC, Triple-Negative Breast Cancer; HER2, Human Epidermal Growth Factor Receptor 2.

The integration of mitochondrial “metabolic signatures” with traditional genomic data represents the next frontier of personalized oncology. Recent advancements in machine learning have allowed for the development of highly accurate diagnostic and prognostic models based on mitochondrial function-related genes (NEMGs) [17,18]. For instance, machine learning-driven 14-gene or 18-gene mitochondrial signatures have demonstrated superior capability in distinguishing tumor from normal tissue and predicting patient survival outcomes with high C-indices [18,19]. As we enter the era of “metabolic-genomic” integration, understanding the interplay between nuclear susceptibility genes (*BRCA1/2*), oncogenic drivers (*MYC*), and mitochondrial bioenergetics is essential for identifying novel therapeutic vulnerabilities [18] (Fig. 1). 

This review aims to synthesize the current research regarding the role of mitochondria in the precision medicine of BC. We examine the molecular mechanisms by which nuclear-encoded genes govern mitochondrial metabolism, the development of metabolic gene signatures as clinical biomarkers, and the therapeutic landscape of targeting mitochondrial vulnerabilities to overcome drug resistance. By bridging the gap between basic metabolic research and clinical application, we outline a potential trajectory for the future of BC precision medicine.

## 2 Fundamental Molecular Mechanisms: Interplay between Nuclear Genes and Mitochondrial Metabolism

**Figure 2:**
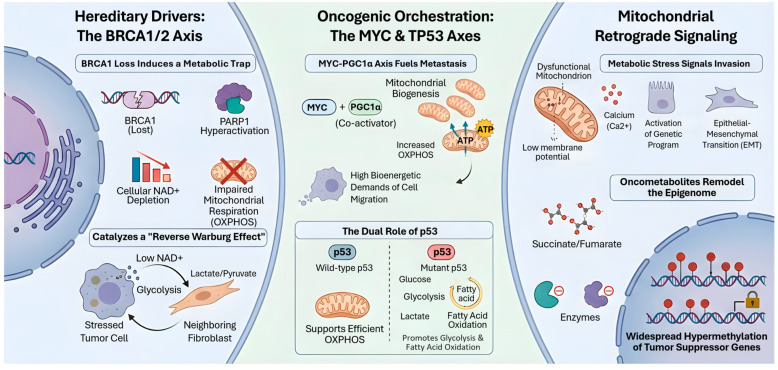
**Molecular mechanisms of metabolic reprogramming and mitochondrial signaling in malignancy.** Hereditary Drivers: The *BRCA1/2* Axis: The loss of BRCA1 function triggers a “metabolic trap” characterized by the hyperactivation of *PARP1*, which leads to severe cellular NAD^+^ depletion. This depletion impairs mitochondrial respiration (OXPHOS), forcing a metabolic shift toward a “Reverse Warburg Effect” where stressed tumor cells utilize lactate and pyruvate provided by neighboring fibroblasts. Abb: BRCA1/2, Breast Cancer Susceptibility Gene 1/2; MYC, Myelocytomatosis; PGC1α, Peroxisome proliferator-activated receptor γ coactivator 1-α; NAD^+^: Nicotinamide Adenine Dinucleotide; PARP1: Poly(ADP-ribose) polymerase 1.

The metabolic identity of BC is not an autonomous feature of the organelle but is strictly governed by the bidirectional communication between the nuclear genome and the mitochondrial network. This mitonuclear crosstalk ensures that mitochondrial bioenergetics are synchronized with the biosynthetic demands of the cell and its environmental stress responses. This section details how hereditary mutations, oncogenic drivers, and retrograde signaling loops converge to reprogram mitochondrial function in BC (Fig. 2).

### 2.1 Hereditary Susceptibility: BRCA1/2 and the NAD^+^/NADH Axis

The *BRCA1* is traditionally characterized as a nuclear tumor suppressor involved in the repair of DNA double strand breaks via homologous recombination. However, contemporary research has identified a significant fraction of phosphorylated BRCA1 protein localized within the mitochondria, specifically associated with mitochondrial nucleoids [20]. This mitochondrial localization is essential for the maintenance of mitochondrial DNA (mtDNA) stability and the regulation of mitophagy, which is the selective autophagic clearance of damaged mitochondria [21]. When BRCA1 function is lost, the mitochondria experience a profound metabolic trap involving the NAD^+^/NADH redox balance. Loss of BRCA1 induces chronic genomic instability, leading to the hyperactivation of Poly(ADP-ribose) polymerase 1 (*PARP1*) as it attempts to repair accumulated nuclear DNA damage [22]. Because *PARP1* utilizes nicotinamide adenine dinucleotide (NAD^+^) as its primary substrate to synthesize poly(ADP ribose) chains, its overactivation causes a systemic depletion of the cellular NAD^+^ pool [22]. This depletion directly impacts the mitochondrial electron transport chain (ETC), specifically Complex I, which requires NAD^+^ as an electron acceptor to convert NADH back into NAD^+^. Consequently, the NAD^+^/NADH ratio drops significantly, leading to impaired OXPHOS and an increased reliance on glycolysis [23].

Furthermore, BRCA1 deficient cells exhibit elevated levels of mitochondrial reactive oxygen species (ROS), particularly hydrogen peroxide (H_2_O_2_), which further stabilizes Hypoxia Inducible Factor 1α (HIF1α) and drives a Reverse Warburg Effect [23]. In this scenario, the oxidative stress within *BRCA1* mutated epithelial cells is transferred to the neighboring tumor stroma, inducing aerobic glycolysis in associated fibroblasts. These fibroblasts then feed the cancer cells with high energy metabolites such as lactate and pyruvate, which can be utilized by the cancer cells to fuel the TCA cycle despite their impaired intrinsic OXPHOS capacity [24]. This metabolic imprinting is a hallmark of the TNBC (basal-like) subtype, where *BRCA1* mutations drive a systemic NAD^+^ depletion trap, distinguishing its metabolic requirements from those of receptor-positive subtypes.

### 2.2 Oncogenic Orchestration: The MYC/PGC1α and TP53 Axes

The *MYC* oncogene acts as a master transcriptional regulator that synchronizes the nuclear and mitochondrial genomes to support rapid biomass accumulation. *MYC* directly promotes the expression of mitochondrial transcription factor A (*TFAM*) and other NEMGs involved in the assembly of the respiratory chain [12,14]. Central to this process is the *PGC1α*, which functions as the primary rheostat for mitochondrial biogenesis. In metastatic BC cells, the *MYC/PGC1α* axis is often upregulated to meet the high bioenergetic demands of migration and colonization [14,25]. Recent research highlights that PGC1α enhances OXPHOS and mitochondrial biogenesis, which is essential for the survival of circulating tumor cells (CTCs) and the formation of distant metastases [26]. While the Warburg Effect suggests that cancer cells move away from mitochondrial respiration, the *MYC/PGC1α* axis demonstrates that aggressive BC cells actually re-adopt OXPHOS to achieve metabolic robustness under microenvironmental stress [25,26]. This metabolic flexibility allows cells to survive in nutrient deprived environments by maximizing ATP yield per molecule of glucose.

The tumor suppressor p53 (*TP53*) plays a dual role in regulating mitochondrial health. Wild type p53 supports mitochondrial efficiency by transcriptionally activating synthesis of cytochrome *c* oxidase 2 (*SCO2*), which is crucial for the assembly of cytochrome *c* oxidase or complex IV [27,28]. It also upregulates *TIGAR* (*TP53* induced glycolysis and apoptosis regulator) to inhibit glycolysis and divert glucose toward the pentose phosphate pathway for NADPH production, thereby maintaining redox homeostasis and limiting ROS damage [28]. In contrast, *TP53* mutations, which are found in over 80% of TNBCs, drastically remodel the mitochondrial landscape. Mutant p53 loses its ability to promote *SCO2* mediated OXPHOS, leading to a shift toward glycolysis [27,29]. Paradoxically, in basal like BCs, mutant p53 has been shown to upregulate the expression of carnitine palmitoyltransferase 1C (*CPT1C*), a key regulator of fatty acid oxidation [30]. This enables mutant p53 cells to utilize lipids as an alternative fuel source, providing a survival advantage during glucose deprivation and contributing to chemoresistance [27]. Furthermore, mutant p53 interacts directly with the mitochondrial inner membrane to alter the permeability transition pore, making cells more resistant to apoptosis induced by mitochondrial stress.

### 2.3 Mitochondrial Retrograde Signaling: From Metabolites to Epigenetics

Mitochondria are active signaling organelles that communicate their functional status to the nucleus via retrograde signaling. In BC, mitochondrial dysfunction, which is characterized by mtDNA depletion or reduced membrane potential, triggers the release of mitochondrial calcium into the cytoplasm [31]. This influx activates the Calcineurin NFAT signaling pathway, which induces the expression of genes associated with the epithelial mesenchymal transition (EMT), such as Snail, Slug, and Vimentin [31,32,33]. This retrograde loop effectively converts metabolic stress into a pro invasive genetic program, generating BC stem cells that are inherently resistant to traditional therapies [31]. Additionally, the accumulation of oncometabolites like succinate and fumarate due to TCA cycle dysregulation acts as a critical signal [34,35]. These metabolites can exit the mitochondria and competitively inhibit alpha ketoglutarate dependent dioxygenases in the nucleus, including the TET family of DNA demethylases and the JmjC domain containing histone demethylases [34,36]. This leads to widespread epigenetic deregulation, specifically the hypermethylation of tumor suppressor promoters, and the stabilization of HIF1α under normoxic conditions [34,37]. Such metabolic epigenetic crosstalk reinforces the aggressive phenotype and ensures that the cancer cell remains in a perpetually glycolytic and invasive state, even in the presence of adequate oxygen. The physical proximity of mitochondria to the nucleus in aggressive BC cells further facilitates this rapid exchange of signaling metabolites and ions. The specific mitochondrial phenotypes and associated therapeutic vulnerabilities for each breast cancer subtype are summarized in Table 1.

**Table 1:** Subtype-specific mitochondrial phenotypes and therapeutic vulnerabilities in breast cancer.

*Subtype*	*Dominant Mitochondrial Phenotype*	*Key Molecular*	*Clinical Context*	*Targeted Vulnerability*
*Luminal (ER+)*	Increased OXPHOS & Biogenesis (via TOMM20)	SIRT3, BCL2	Endocrine Resistance	BH3 Mimetics (Venetoclax)
*HER2-Enriched*	Metabolic Flexibility (Glycolysis to OXPHOS)	HER2 signaling	Adjuvant Therapy	Complex I Inhibition
*TNBC (Basal-like)*	Glycolytic/Glutaminolytic Dominance; Mitonuclear Stress	MYC, TP53 (Mutant), BRCA1 (Loss)	Chemoresistance & Metastasis	Synthetic Lethality (MYC + Complex I; PARPi + Mito-stress)

## 3 Precision Diagnosis: Developing Metabolic Markers Based on Genetic Screening

**Figure 3:**
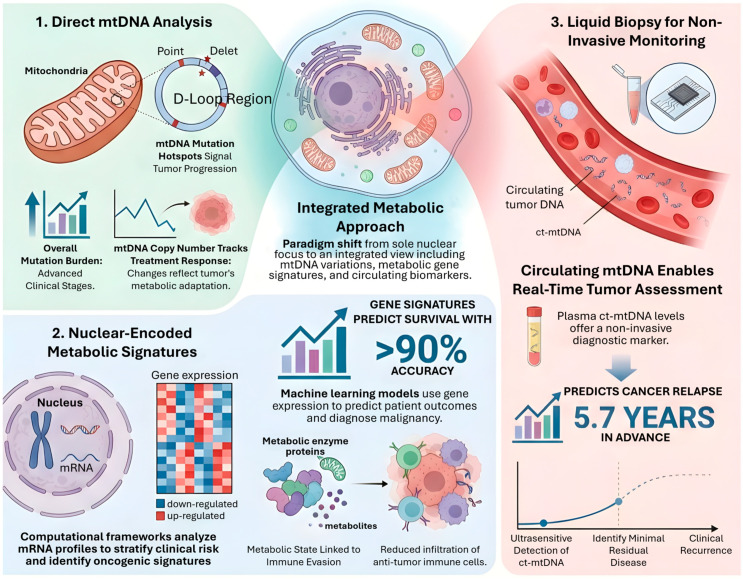
**Transition from nuclear-focused diagnostics to an integrated metabolic approach for cancer prognosis.** This paradigm combines Direct mtDNA Analysis of the D-loop region and copy number with Nuclear-Encoded Metabolic Signatures to stratify clinical risk. Computational frameworks and machine learning models utilize these multi-omic data streams to identify immune evasion and predict patient survival with over 90% accuracy. Furthermore, the application of Liquid Biopsy for ct-mtDNA allows for non-invasive, ultrasensitive detection of minimal residual disease.

The core of precision medicine in BC lies in the development of biomarkers with high sensitivity and specificity. As high throughput sequencing technologies have advanced, the focus of research has expanded from the nuclear genome to the mitochondrial genome and its associated metabolic signatures [9,38,39]. This section explores how mitochondrial DNA variations, nuclear encoded metabolic features, and liquid biopsy technologies are constructing a new paradigm for the precision diagnosis of BC (Fig. 3).

### 3.1 Mitochondrial DNA Heteroplasmy Screening

The mitochondrial genome or mtDNA is highly susceptible to damage due to its proximity to the site of reactive oxygen species production and its lack of protective histones and robust repair mechanisms. In BC, mtDNA mutations often exhibit heteroplasmy, which refers to the co-existence of mutant and wild type mitochondrial genomes within a single cell. The dynamic shift in heteroplasmy levels is recognized as a significant driver of tumor evolution and metabolic adaptation. Research has established that the D loop region, a non-coding displacement loop, is a mutation hotspot in BC mtDNA [40]. This region is critical because it contains essential regulatory elements for the replication and transcription of the entire mitochondrial genome. Specific mutations in the D loop, such as the T16189C and A10398G variants, have been strongly correlated with increased BC susceptibility and malignant progression [41]. Large scale analyses of clinical samples demonstrate that the burden of mtDNA mutations increases in direct correlation with the clinical stage of the disease. Specifically, patients in stage III and stage IV exhibit significantly higher levels of mitogenomic diversity compared to those in early stages, suggesting that these variations provide a selective advantage to tumor cells operating under severe microenvironmental stress [17].

In addition to point mutations, mitochondrial DNA copy number or mtDNAcn serves as a quantitative indicator of mitochondrial mass and bioenergetic capacity. In TNBC, it has been observed that tumor tissues frequently exhibit lower mtDNAcn relative to adjacent normal tissues. However, in cases where residual tumor cells survive neoadjuvant chemotherapy, there is often a compensatory resurgence in mtDNAcn [42]. This ‘compensatory resurgence’ is quantitatively defined as a statistically significant increase in mtDNAcn within chemoresistant clones, typically represented by a ≥1.5-fold increase relative to pre-treatment biopsies or the restoration of mtDNA levels to match adjacent normal tissues [42]. The scientific rationale for this threshold lies in the metabolic plasticity of surviving cells, which must upregulate mitochondrial mass to compensate for drug-induced oxidative stress and fulfill the high energetic demands of therapeutic escape. To ensure the rigor of these measurements, clinical studies utilize normalized dPCR or qPCR assays, where mtDNAcn is calculated relative to nuclear reference genes to account for variations in cell number and DNA quality. This recovery reflects the metabolic plasticity of the cells as they adapt to therapeutic pressure, positioning mtDNAcn as a valuable biomarker for monitoring treatment response and potential recurrence.

### 3.2 Metabolic Gene Signatures Derived from Nuclear Encoded Mitochondrial Genes

While mtDNA mutations are primary indicators, the metabolic phenotype of a tumor is largely governed by nuclear encoded mitochondrial genes or NEMGs. Integrating large scale genomic datasets from sources such as The Cancer Genome Atlas or TCGA and the Gene Expression Omnibus or GEO allows for the identification of metabolic gene signatures with high prognostic value. The development of these signatures typically employs advanced bioinformatics pipelines and machine learning algorithms, including Least Absolute Shrinkage and Selection Operator or LASSO regression and random forest models. To ensure model stability and reproducibility, these frameworks utilize rigorous hyperparameter optimization; for instance, LASSO regression models frequently employ 10-fold cross-validation to identify the optimal penalty parameter that minimizes partial likelihood deviance. Similarly, random forest models used in these signatures typically specify a standardized number of trees (ntree = 500) and a fixed number of variables randomly sampled at each split (mtry) to prevent overfitting and ensure consistent feature importance rankings across diverse datasets. For instance, recent studies have validated a 14 gene mitochondrial related prognostic signature that can accurately predict overall survival at one, three, and five year intervals with an area under the curve or AUC exceeding 0.90 in external validation cohorts [19,43]. Another 18-gene diagnostic model developed in 2025 through the evaluation of over 113 machine learning combinations demonstrated a 0.947 accuracy rate in differentiating between malignant and healthy breast tissues [18]. These metabolic panels provide more than just survival predictions. They also offer insights into the tumor microenvironment. Higher expression levels of certain NEMGs are associated with reduced infiltration of natural killer cells and T cells, implying that a more oxidative metabolic state may facilitate immune evasion by the tumor [44,45]. By utilizing these metabolic fingerprints, clinicians can classify patients into specific metabolic subtypes that traditional genomic subtyping might overlook, enabling the delivery of more targeted interventions.

### 3.3 Potential of Liquid Biopsy Applications

Liquid biopsy technology offers a non-invasive method to monitor the metabolic evolution of BC in real time. Circulating tumor mitochondrial DNA or ct-mtDNA has emerged as a promising marker for early diagnosis and the detection of minimal residual disease or MRD. Ct-mtDNA consists of fragmented mitochondrial DNA released into the systemic circulation by necrotic or apoptotic tumor cells. Clinical evidence confirms that the concentration of ct-mtDNA in the plasma of BC patients is significantly higher than in healthy controls [46]. Beyond simple quantification, ct-mtDNA allows for the real time assessment of the tumor metabolic state without the need for repetitive tissue biopsies. One of the most significant applications of ct-mtDNA is in monitoring MRD following surgical intervention. Ultrasensitive sequencing techniques can detect MRD signals in the blood years before a clinical recurrence is visible through standard imaging. In one cohort study, ct-mtDNA detection anticipated late relapse in early-stage BC by an average of 5.7 years [47]. This comprehensive approach provides a dynamic molecular portrait of the disease, allowing for early intervention with metabolic therapies before the transition to macro metastatic recurrence occurs. However, the clinical translation of these metabolic indicators requires the rigorous validation of assays across diverse patient populations and the establishment of clear regulatory guidelines [4]. Current technical challenges involve the need for standardized detection protocols for ct-mtDNA to ensure consistency in quantification and sensitivity across different laboratory settings [46]. The transition to metabolic markers in the clinical setting requires the standardization of detection protocols and the validation of these markers in diverse patient populations. However, the current trajectory of research indicates that mitochondrial and metabolic signatures may serve as valuable adjuncts to the next generation of BC diagnostics, pending further clinical validation.

### 3.4 Technical Hurdles and Methodological Standardization

The translation of mitochondrial metabolic indicators into standardized clinical practice requires the rigorous validation of assays across diverse patient populations and the establishment of clear regulatory guidelines [4]. Current technical challenges involve the need for standardized detection protocols for ct-mtDNA to ensure consistency in quantification and sensitivity across different laboratory settings [46]. Furthermore, the reliable detection of minimal residual disease (MRD) through liquid biopsy remains dependent on addressing critical pre-analytical variables, including specimen type, tumor fraction, and heteroplasmy thresholds, to ensure that low-frequency mitochondrial signals are distinguishable from biological background noise [46,47]. In the context of nuclear-encoded mitochondrial gene (NEMG) signatures, the application of machine learning necessitates strategies to account for inter-platform variability and batch effects that can adversely impact the reproducibility of prognostic models [17,18]. Developing and implementing robust normalization strategies is therefore a prerequisite for the clinical deployment of these metabolic panels [18]. Ultimately, the transition to metabolic precision medicine is contingent upon large-scale prospective validation and the creation of regulatory frameworks that address the significant costs and accessibility of multi-omics integration [4].

## 4 Clinical Targeted Therapy: Precise Attacks on Mitochondrial Vulnerabilities

**Figure 4:**
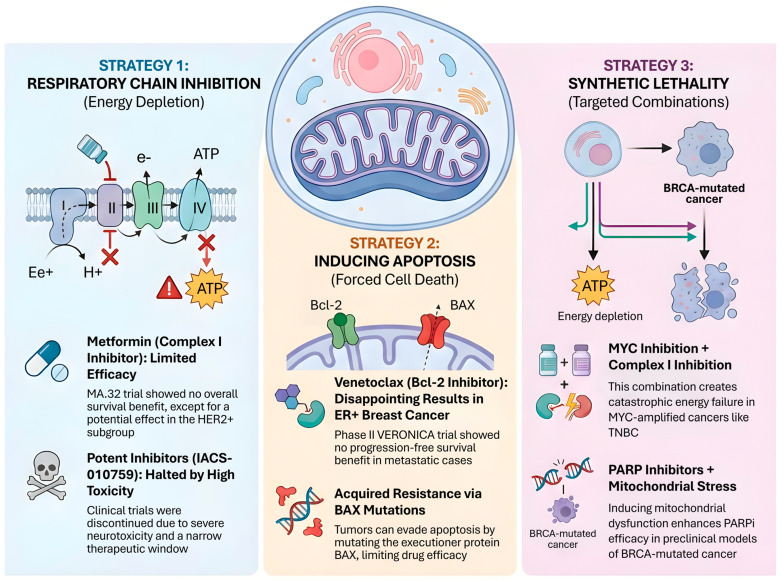
**Therapeutic strategies and clinical outcomes targeting mitochondrial metabolism.** Current clinical approaches focus on Strategy 1: Respiratory Chain Inhibition, where Complex I inhibitors like Metformin showed no overall survival benefit in the MA.32 trial, and potent alternatives like IACS-010759 were halted due to high neurotoxicity; Strategy 2: Inducing Apoptosis, exemplified by Venetoclax, which failed to improve outcomes in the Phase II VERONICA trial for metastatic ER^+^ BC due to acquired resistance such as *BAX* mutations; and Strategy 3: Synthetic Lethality, utilizing combinations like *MYC* and Complex I inhibition to induce energy failure or pairing PARP inhibitors with mitochondrial stress to enhance efficacy in BRCA-mutated cancers. Abb: BAX, Bcl-2 associated X protein; Bcl-2, B cell lymphoma 2; TNBC, Triple-Negative Breast Cancer.

The transition of mitochondrial research from basic biochemistry to clinical application has led to the identification of several druggable vulnerabilities within the organelle. These targets include the electron transport chain components, the apoptotic machinery, and metabolic enzymes involved in synthetic lethal relationships. This section reviews the current status of mitochondrial targeted therapies in BC, emphasizing clinical trial outcomes and emerging strategies (Fig. 4).

### 4.1 Respiratory Chain Complex Inhibitors

The ETC is the primary site of ATP production via OXPHOS. Inhibiting specific complexes within the ETC represents a strategy to starve cancer cells of energy and biosynthetic precursors.

#### 4.1.1 Metformin and Complex I Inhibition

Metformin, a biguanide primarily used for type 2 diabetes, has garnered significant interest in oncology due to its ability to inhibit Mitochondrial Complex I. The mechanism involves the transient and non-competitive inhibition of the NADH dehydrogenase enzyme within Complex I, which leads to a decrease in the proton gradient across the inner mitochondrial membrane and a reduction in ATP production [14]. Systemically, metformin lowers circulating insulin and insulin like growth factor 1 (IGF 1) levels, which are known mitogens for BC cells. Locally, the resulting increase in the AMP to ATP ratio activates the AMP-activated protein kinase (AMPK) pathway, subsequently inhibiting the mammalian target of rapamycin (mTOR) signaling and protein synthesis [14,48,49].

The most definitive evaluation of metformin in the adjuvant setting for BC was the MA.32 trial. This phase III randomized double-blind trial enrolled 3649 non-diabetic patients with high risk early-stage BC [50]. The primary analysis published in 2022 revealed that the addition of metformin to standard therapy did not significantly improve invasive disease free survival (IDFS) or overall survival (OS) in the overall population of hormone receptor positive or negative patients [50]. However, a significant subgroup effect was observed in patients with HER2 positive BC. In this cohort, metformin was associated with a lower risk. This subgroup was selected based on the distinct mitochondrial dependencies of HER2-enriched tumors. However, as this was a secondary analysis involving multiple comparisons, the findings are interpreted as hypothesis-generating rather than definitive, necessitating prospective validation to ensure statistical reproducibility [50]. Secondary analyses in 2023 further confirmed that metformin did not reduce the risk of new primary non BCs in this population, suggesting that its role as a broad spectrum chemopreventive agent in non-diabetic BC patients is limited [51].

#### 4.1.2 Novel Complex I Inhibitors and the IACS 010759 Challenge

Beyond metformin, more potent and selective Complex I inhibitors have been developed to achieve greater metabolic suppression. IACS 010759 is a small molecule inhibitor designed to target OXPHOS dependent tumors. While preclinical models showed robust antitumor activity, clinical progression has been hindered by safety concerns. Phase I dose escalation trials (NCT02882321 and NCT03291938) in patients with advanced solid tumors and acute myeloid leukemia were discontinued in 2023 due to a narrow therapeutic index [52]. Patients experienced significant treatment emergent adverse events, including lactic acidosis and neurotoxicity, such as peripheral neuropathy and visual impairment [52,53]. These toxicities arose because the brain and peripheral nerves are highly sensitive to mitochondrial inhibition, leading to a failure to maintain target drug exposure without severe side effects [53]. These findings highlight the critical challenge of balancing efficacy with the systemic necessity of mitochondrial function in healthy tissues.

### 4.2 Inducing the Mitochondrial Apoptosis Pathway: BH3 Mimetics

Mitochondria play a central role in the intrinsic apoptotic pathway, which is regulated by the B cell lymphoma 2 (Bcl-2) family of proteins. Many BCs, particularly estrogen receptor (ER) positive subtypes, overexpress anti apoptotic proteins like Bcl-2 to evade cell death induced by oncogenic stress or chemotherapy.

#### Venetoclax in BCL 2 Positive BC

Venetoclax is a selective BH3 mimetic that inhibits Bcl-2 by binding to its BH3 binding groove, thereby displacing pro-apoptotic proteins like BAX and BAK to trigger cytochrome *c* release and subsequent apoptosis [54]. Since *Bcl-2* is overexpressed in approximately 85 percent of ER^+^ BCs, it represents a high value target in this population [55]. Clinical trials have explored the combination of Venetoclax with endocrine therapy. In the VERONICA phase II trial, the addition of Venetoclax to fulvestrant did not improve progression free survival (PFS) in patients with ER^+^, HER2^−^ metastatic BC who had previously progressed on CDK4/6 inhibitors [56]. However, early phase studies such as the PALVEN trial (NCT03900884), which combines Venetoclax with letrozole and palbociclib, are ongoing with an estimated completion in late 2025 [57]. Recent translational research has identified emergent *BAX* mutations in the blood of BC patients treated with Venetoclax, suggesting that tumor cells can develop resistance by mutating the executioner of apoptosis [55]. This underscores the need for precision functional approaches to monitor and overcome adaptive resistance.

### 4.3 Synthetic Lethality strategies

Synthetic lethality occurs when the simultaneous disruption of two pathways leads to cell death, while the disruption of either alone is compatible with survival. In the context of mitochondrial metabolism, this involves targeting compensatory pathways or specific genetic dependencies.

#### 4.3.1 MYC Inhibition and Complex I Vulnerability

One of the most significant breakthroughs in 2025 was the identification of a synthetic lethal relationship between the *MYC* oncogene and mitochondrial complex I. Using a genome wide CRISPR screen, researchers discovered that inhibiting *MYC* triggers a compensatory upregulation of Complex I genes to maintain cellular bioenergetics [58]. When *MYC* inhibition is paired with a Complex I inhibitor, the cancer cell can no longer compensate for the loss of both glycolytic and oxidative fluxes, leading to catastrophic energy failure and increased infiltration of CD8^+^ T cells [58]. This strategy is particularly relevant for aggressive TNBCs where *MYC* is frequently amplified.

#### 4.3.2 PARP Inhibitors and Mitochondrial Stress

For patients with BRCA1/2 mutations, PARP inhibitors (PARPi) are the standard of care. Emerging research suggests that combining PARPi with mitochondria targeted gene therapy can enhance treatment outcomes in TNBC. For example, the cmLumiOpto system directly disrupts the mitochondrial membrane potential and induces ROS production [59]. In preclinical models, this combination achieved a 95 to 100 percent reduction in tumor burden and successfully inhibited metastasis. By inducing mitochondrial dysfunction, these therapies create a state of high metabolic stress that sensitizes cells to the DNA damaging effects of PARPi, representing a novel metabolic synthetic lethality [59]. Furthermore, mitochondrial dysfunction has been shown to play a critical role in the synergistic cytotoxic effects of specific drug combinations [60], and targeting mitochondrial metabolism is increasingly recognized as a vital strategy to overcome hormone resistance in breast cancer [61]. In summary, while targeting mitochondria offers a logical approach to overcome drug resistance, the clinical translation remains complex. The failure of broad-spectrum inhibitors like IACS 010759 suggests that the future of mitochondrial therapy lies in patient selection through metabolic subtyping and the use of combination strategies that exploit specific genetic vulnerabilities.

## 5 Metabolic Plasticity and Drug Resistance: Molecular Roots of Clinical Challenges

**Figure 5:**
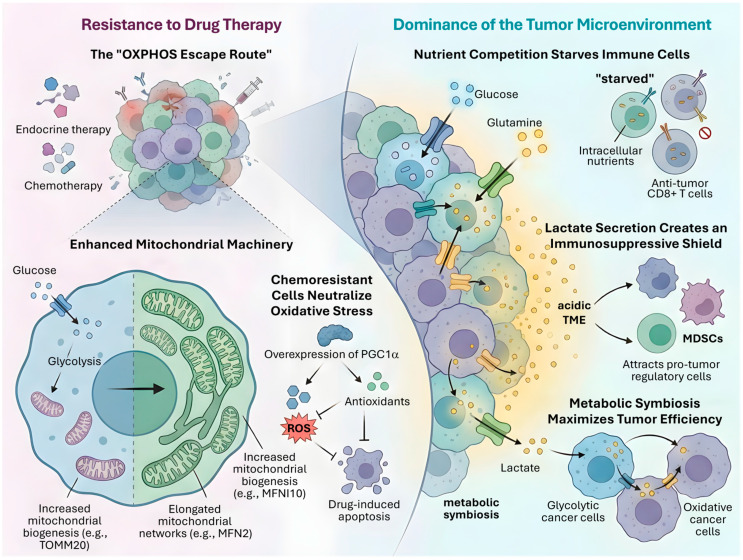
**The “OXPHOS Escape Route” and metabolic dominance within the tumor microenvironment.** BC cells evade endocrine and chemotherapy by enhancing mitochondrial machinery, including increased biogenesis (via TOMM20) and the formation of elongated mitochondrial networks (via MFN2). This metabolic shift is often driven by the overexpression of *PGC1α*, which neutralizes drug-induced oxidative stress by increasing antioxidant production, thereby inhibiting apoptosis. Within the tumor microenvironment (TME), aggressive nutrient competition for glucose and glutamine “starves” anti-tumor CD8^+^ T cells, leading to immune exhaustion. Simultaneously, high levels of lactate secretion create an acidic TME that acts as an immunosuppressive shield, attracting pro-tumor regulatory cells like MDSCs. This landscape facilitates a metabolic symbiosis, where glycolytic cancer cells export lactate to be utilized as fuel by oxidative cancer cells, maximizing overall tumor efficiency and survival.

The clinical trajectory of BC is often compromised by the emergence of therapeutic resistance, a process deeply rooted in the metabolic plasticity of tumor cells. This adaptability allows cancer cells to survive extreme physiological stress by reconfiguring their bioenergetic and biosynthetic networks. Unlike healthy tissues that maintain metabolic homeostasis, BC cells exhibit a high degree of flexibility, shifting between glycolysis and OXPHOS in response to pharmacological blockade. This section examines the molecular mechanisms of metabolic rewiring that underpin resistance to endocrine therapy, chemotherapy, and the suppressive nature of the tumor microenvironment (Fig. 5).

### 5.1 Endocrine and Chemotherapy Resistance: The OXPHOS Escape Route

Endocrine therapy resistance is a major obstacle in the management of ER positive BC. While agents like tamoxifen and aromatase inhibitors successfully disrupt estrogen signaling, a subset of cancer cells survives by undergoing a profound metabolic shift. These resistant cells frequently exhibit a transition from a glycolytic phenotype to one characterized by high mitochondrial activity and increased OXPHOS capacity [15]. Recent studies published in 2025 have confirmed that this metabolic conversion is driven by the upregulation of mitochondrial biogenesis markers such as the translocase of outer mitochondrial membrane 20 or *TOMM20* and the SIRT3 protein [7]. High mitochondrial mass in these cells provides the necessary energy to bypass the growth inhibitory effects of estrogen deprivation, effectively facilitating a state of metabolic persistence.

In the context of chemotherapy, metabolic plasticity enables the survival of drug tolerant persister cells. These cells often exit the cell cycle and adopt a dormant state that is less sensitive to traditional cytotoxic agents like paclitaxel or doxorubicin. Research indicates that the survival of these persisters is critically dependent on mitochondrial respiration and fatty acid oxidation [58,62]. In these aggressive subtypes, mutant *TP53* has been shown to upregulate the expression of *CPT1C*, enabling cancer cells to utilize lipids as an alternative fuel source during glucose deprivation [30]. In TNBC, chemoresistant clones have been shown to overexpress *PGC1α*, which not only enhances OXPHOS but also increases the production of antioxidants like glutathione [63]. This dual function allows the cells to maintain energy production while neutralizing the reactive oxygen species generated by chemotherapeutic stress, thereby preventing apoptosis.

Furthermore, mitochondrial dynamics, specifically the balance between mitochondrial fusion and fission, play a key role in resistance. Resistant BC cells often exhibit an elongated mitochondrial morphology promoted by fusion proteins like MFN2. This elongated network is more efficient at producing ATP and sequestering pro apoptotic factors, thereby raising the threshold for mitochondrial outer membrane permeabilization and cell death [64]. Targeting these structural adaptations represents a potential strategy to re sensitize resistant tumors to conventional therapies. These structural adaptations, particularly MFN2-mediated fusion, often exhibit subtype-specific characteristics [64]. While MFN2 facilitates the formation of elongated networks for energy production and resistance, this dynamic has been identified as a critical therapeutic target specifically in TNBC [65]. Such findings underscore the distinct mitochondrial bioenergetic profiles observed between Luminal and Basal subtypes, which dictate their unique metabolic escape routes during therapy.

### 5.2 Metabolic Competition in the Tumor Microenvironment

The tumor microenvironment or TME is a site of intense competition for limiting nutrients, where the metabolic needs of the cancer cell directly conflict with those of infiltrating immune cells. Aggressive BC cells utilize aerobic glycolysis at a high rate, a process that depletes the local glucose supply and generates a large volume of lactate [26,66]. This glucose deprivation is particularly detrimental to CD8^+^ T cells and natural killer cells, which require rapid glycolytic flux to fuel their effector functions, such as the secretion of perforin and granzyme B. Beyond glucose, the competition for amino acids like glutamine and fatty acids is a critical factor in immune evasion [67]. Glutamine is essential for both cancer cell proliferation and T cell activation. In the TME of TNBC, cancer cells often overexpress glutamine transporters like ASCT2, effectively starving the T cells and inducing a state of metabolic exhaustion [18]. Additionally, the accumulation of lactate leads to the acidification of the TME, with pH levels often dropping below 6.5. This acidic environment inhibits the mammalian target of rapamycin or mTOR signaling in T cells while promoting the recruitment and activation of myeloid derived suppressor cells or MDSCs and regulatory T cells or Tregs [68,69,70]. The spatial distribution of nutrients further complicates this competition. In poorly vascularized regions of the tumor, hypoxia induces the expression of HIF1α in both cancer and immune cells. However, cancer cells adapt more effectively by switching to alternative metabolic pathways, such as acetate or ketone body utilization, which immune cells are less equipped to exploit [65]. Metabolic escape routes differ significantly across subtypes. While ER^+^ BCs often shift toward an OXPHOS-high phenotype via *TOMM20* upregulation to evade endocrine therapy [15], TNBC cells frequently exhibit a more glycolytic and glutaminolytic dominance to thrive in the nutrient-deprived tumor microenvironment [18]. This metabolic plasticity allows TNBC to effectively outcompete infiltrating immune cells for essential amino acids like glutamine. This metabolic dominance allows the tumor to create a sanctuary where it can proliferate shielded from the host immune system.

### 5.3 Mechanisms of Metabolic Switching and Symbiosis

When primary metabolic pathways are pharmacologically targeted, BC cells demonstrate a remarkable ability to activate compensatory loops. This phenomenon, known as metabolic switching, is a fundamental cause of the failure of metabolic monotherapies. For instance, the inhibition of glycolysis through lactate dehydrogenase A or LDHA targeting often leads to a rapid increase in mitochondrial glutaminolysis and fatty acid oxidation to maintain the TCA cycle flux [71,72]. A particularly sophisticated form of this plasticity is metabolic symbiosis between different cell populations within the tumor. In many BCs, glycolytic cells located in hypoxic regions export lactate via the monocarboxylate transporter 4 or MCT4. This lactate is then taken up by oxidative cancer cells in well oxygenated regions through MCT1, where it is converted back to pyruvate to fuel the TCA cycle [73,74]. To accurately distinguish these metabolic subpopulations within the complex tumor architecture, researchers utilize spatial transcriptomics and single-cell RNA sequencing, which enable the visualization of metabolic niches based on the expression of markers such as *LDHA* and *MCT4* for glycolytic cells versus *TOMM20* and *MCT1* for oxidative cells. These high-resolution approaches effectively exclude stromal cell contamination (such as cancer-associated fibroblasts) by employing cell type specific markers or bioinformatic deconvolution to isolate the malignant epithelial transcriptome for independent metabolic flux analysis. This adaptive rewiring allows tumor cells to bypass the glycolytic blockade by utilizing alternative carbon sources to maintain energy production and biosynthetic requirements. This symbiotic relationship allows the tumor as a whole to maximize nutrient utility and thrive under heterogeneous conditions.

This switching is coordinated by global metabolic sensors such as the AMP activated protein kinase or AMPK and the mTOR complex. Under conditions of energy stress, AMPK is activated to inhibit biosynthetic processes and stimulate catabolic pathways like mitophagy and fatty acid oxidation [65,75]. Conversely, if the cell is supplied with abundant nutrients, mTOR stimulates the production of proteins and lipids required for cell division. The crosstalk between these sensors ensures that the cancer cell can pivot its metabolic strategy almost instantaneously upon sensing a change in nutrient availability or therapeutic pressure. Furthermore, recent evidence suggests that BC cells can acquire mitochondria from neighboring stromal cells through tunneling nanotubes or extracellular vesicles. This horizontal mitochondrial transfer allows cancer cells with damaged or inhibited mitochondria to restore their OXPHOS capacity and survive metabolic crises [76,77]. Such mechanisms of extreme plasticity underscore the need for multi target metabolic inhibitors that can block both the primary pathways and the potential escape routes used by the tumor.

## 6 Future Prospects: Artificial Intelligence (AI) Assisted Metabolic Modeling and Next Generation Screening

**Figure 6:**
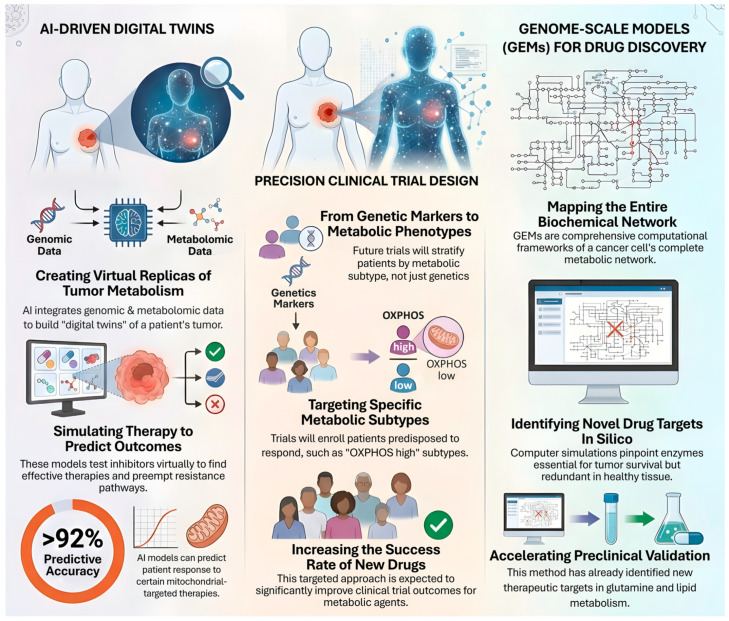
**Future directions in precision medicine utilizing computational frameworks to predict therapeutic response and discover novel targets.** The next generation of oncology integrates AI-Driven Digital Twins where genomic and metabolomic data streams are merged to create high-fidelity virtual replicas of a patient’s tumor metabolism. These models simulate various inhibitors virtually to find effective therapies and predict outcomes with over 92% accuracy. Precision Clinical Trial Design is shifting from static genetic markers to dynamic Metabolic Phenotypes by stratifying patients into subgroups such as “OXPHOS high” to enhance the likelihood of clinical efficacy for new metabolic agents. Furthermore, Genome-Scale Models (GEMs) provide a comprehensive computational map of the entire biochemical network to identify essential enzymes that are redundant in healthy tissue but critical for tumor survival. This integrated computational approach has already accelerated preclinical validation by pinpointing novel therapeutic targets within glutamine and lipid metabolism.

The integration of AI and systems biology into the clinical workflow represents the next logical step in the evolution of BC precision medicine. As the volume of multi-omics data increases, traditional statistical methods are often insufficient to capture the dynamic and non-linear nature of metabolic networks. This section explores how computational modeling and advanced machine learning architectures are being used to predict patient specific metabolic vulnerabilities and refine clinical trial designs (Fig. 6).

### 6.1 Integrated Genomic Metabolomic Modeling and Digital Twins

While recent AI models trained on patient-derived organoid data have reported predictive accuracies exceeding 92%, it is essential to evaluate these findings within the context of model interpretability and clinical calibration [18,36]. To ensure the reliability of these predictions and mitigate the risk of model overfitting, these frameworks typically utilize a 10-fold cross-validation strategy, often repeated across 100 iterations to stabilize performance metrics and provide a robust estimate of model generalizability. In specific instances involving smaller or highly specialized patient cohorts, Leave-One-Out Cross-Validation (LOOCV) is employed to maximize the utility of the available training data. The high performance of mitochondrial gene signatures often relies on retrospective datasets where cohort sizes may be limited, potentially increasing the risk of overfitting and feature leakage if rigorous cross-validation and external validation are not employed [19,40]. Furthermore, the transition of these models into clinically realistic workflows requires addressing the “black box” nature of deep learning through interpretability frameworks to ensure clinicians can understand the biological rationale behind AI-generated insights [17,18]. Current studies frequently lack prospective testing in real-world settings, which remains a critical barrier to establishing the true clinical utility and reliability of metabolic digital twins [4].

### 6.2 Precision Clinical Trial Design Based on Metabolic Phenotypes

A significant limitation of current clinical trials in BC is the reliance on protein expression or genetic mutations as the sole inclusion criteria. The failure of broad spectrum mitochondrial inhibitors in early trials highlights the need for a more nuanced approach to patient selection. Future clinical trials should incorporate metabolic phenotyping as a central component of their design [4]. The novelty of integrating AI-driven metabolic modeling lies in its ability to refine clinical trial selection by shifting inclusion criteria from static genetic markers to dynamic metabolic phenotypes. By utilizing the metabolic gene signatures and liquid biopsy markers discussed in previous sections, researchers can stratify patients into metabolic subtypes such as OXPHOS high or glycolytic high. This stratification ensures that metabolic therapies are tested in populations that are biologically predisposed to respond. For instance, a trial targeting Mitochondrial Complex I would specifically enroll patients whose tumors show high dependence on oxidative phosphorylation and low metabolic flexibility [14]. This paradigm shift in trial design is hypothesized to potentially improve the success rate of metabolic agents in the clinical pipeline by refining patient selection.

### 6.3 Role of Genome Scale Metabolic Models in Drug Discovery

Genome scale metabolic models or GEMs provide a comprehensive computational framework for analyzing the entire biochemical network of a cancer cell. GEMs like Recon3D and the more recent Recon4D integrate thousands of metabolic reactions and gene associations into a single mathematical matrix [78]. In the context of BC, GEMs are used to perform *in silico* gene essentiality screens to identify novel therapeutic targets. By simulating the metabolic requirements for biomass production in specific BC subtypes, GEMs can pinpoint enzymes that are essential for tumor survival but redundant in healthy tissues [79]. This approach has already led to the identification of several non-obvious targets in glutamine and lipid metabolism that are currently entering preclinical validation. As GEMs become more personalized through the incorporation of patient specific data, they will serve as indispensable tools for the development of the next generation of metabolic precision therapies.

## 7 Toward a New ERA of Metabolic Precision Medicine

The integration of mitochondrial metabolism into the precision medicine framework marks a significant departure from the genomic centric approach that has dominated BC research for the past two decades. This review has synthesized the evidence demonstrating that the triangular relationship between nuclear genetic alterations, mitochondrial bioenergetics, and the tumor microenvironment is increasingly recognized as a significant contributor to clinical outcomes. We have explored how hereditary mutations in genes like *BRCA1* and the overactivation of oncogenes like *MYC* serve as blueprints for mitochondrial reprogramming. These genetic drivers do not act in isolation but rather coordinate a complex metabolic network that facilitates tumor growth, metastasis, and the evasion of standard therapies. The development of metabolic gene signatures and the application of liquid biopsy for ct-mtDNA monitoring represent promising tools for translating these basic insights into clinical diagnostics. However, several challenges remain. The translation of metabolic indicators into standardized clinical practice requires the rigorous validation of assays across diverse patient populations and the establishment of clear regulatory guidelines for metabolic biomarkers. Furthermore, the inherent toxicity of targeting mitochondrial pathways that are shared with healthy tissues necessitates the development of more selective inhibitors and sophisticated delivery systems.

## 8 Conclusion

In conclusion, the shift toward a metabolic genomic paradigm offers a powerful strategy to address the enduring challenges of therapeutic resistance and tumor heterogeneity in BC. By leveraging AI to model the dynamic metabolic landscape of individual patients and designing clinical trials that respect the metabolic identity of the tumor, we can move closer to the goal of achieving durable and personalized responses for all patients. Nevertheless, several hurdles remain for the full implementation of metabolic precision medicine. These include the significant cost of multi-omics integration, the need for increased accessibility to advanced metabolomic platforms in clinical settings, and the establishment of clear regulatory frameworks for validating metabolic-based diagnostic signatures.

## Data Availability

Not applicable.

## Abbreviations

AI	Artificial Intelligence
AMPK	AMP-activated protein kinase
AUC	Area under the Curve
BAX	Bcl-2 associated X protein
BC	Breast Cancer
Bcl-2	B cell lymphoma-2
BRCA1	Breast Cancer 1
BRE	Bloom-Richardson-Elston grades
CPT1C	Carnitine Palmitoyltransferase 1c
ct-mtDNA	Circulating tumor Mitochondrial DNA
EMT	Epithelial-Mesenchymal Transition
ER	Estrogen Receptor
ETC	Electron Transport Chain
GEMs	Genome-Scale metabolic Models
GEO	Gene Expression Omnibus
HER2	Human Epidermal Growth Factor Receptor 2
HIF1α	Hypoxia Inducible Factor 1 alpha
IDFS	Invasive Disease-Free Survival
IGF-1	Insulin-like Growth Factor 1
LASSO	Least Absolute Shrinkage and Selection Operator
LDHA	Lactate Dehydrogenase A
LOOCV	Leave-One-Out Cross-Validation
MCT1	Monocarboxylate Transporter 1
MCT4	Monocarboxylate Transporter 4
MDSCs	Myeloid Derived Suppressor Cells
MRD	Minimal Residual Disease
mtDNA	Mitochondrial DNA
mtDNAcn	Mitochondrial DNA Copy Number
mTOR	Mammalian Target Of Rapamycin
NAD^+^	Nicotinamide Adenine Dinucleotide
NEMGs	Nuclear-Encoded Mitochondrial Genes
OS	Overall Survival
OXPHOS	Oxidative Phosphorylation
PARP1	Poly(ADP-ribose) polymerase 1
PFS	Progression-Free Survival
PGC1α	Peroxisome proliferator-activated receptor gamma coactivator 1α
ROS	Reactive Oxygen Species
SCO2	Synthesis of Cytochrome c oxidase 2
TCA	Tricarboxylic Acid cycle
TCGA	The Cancer Genome Atlas
TFAM	Mitochondrial Transcription Factor A
TIGAR	TP53 induced glycolysis and apoptosis regulator
TME	Tumor Microenvironment
TNBC	Triple-Negative Breast Cancer
TNM	Tumor-Node-Metastasis staging system
TOMM20	Translocase of Outer Mitochondrial Membrane 20
TP53	Tumor protein p53
